# P62. Effectiveness of melatonin, IL-25 and siRNA IL-17B in growth control of breast cancer cell lines

**DOI:** 10.1186/2051-1426-2-S2-P36

**Published:** 2014-03-12

**Authors:** G Bottaro Gelaleti, C Leonel, BV Perassi-Jardim, LC Ferreira, MG Moschetta, TF Borin, LB Maschio, GR Martins, AM Viloria-Petit, DAPC Zuccari

**Affiliations:** 1 Universidade Estadual Paulista "Julio de Mesquita Filho", Biologia - Genética, São José do Rio Preto SP, Brazil; 2 Faculdade de Medicina - FAMERP, Biologia Molecular, São José do Rio Preto SP, Brazil; 3 University of Guelph, Biomedical Sciences, Guelph, Canada

## 

Interleukin (IL)-25, a cytokine active in inflammatory processes and production of proinflammatory cytokines, is secreted by mammary epithelial non-malignant cells and induces apoptosis in tumour cells by differential expression of its receptor IL-17RB, highly expressed in malignant cells and lower expressed in non-malignant epithelial cells. It is known that another ligand (IL-17B) compete for the same site of action in tumour cells, contributing to its tumorigenic potential. The melatonin hormone acts in several activities, among them the immune system regulation, proliferation, differentiation and apoptosis process in tumour cells. The aim of this study was to evaluate the effects of treatment with IL-25, silencing gene (siRNA) of cytokine IL-17B and melatonin in human mammary tumour cells for the control of cell proliferation and induction of apoptosis. Non-metastatic mammary tumour cells (MCF-7), metastatic (MDA-MB-231) and a human normal mammary epithelial cells (MCF-10A) were cultured and divided into five treatment groups: group I control, group II treated with IL-25 protein, group III treated with siRNA IL-17B, group IV treated with melatonin and group V joint treatments. The gene silencing was standardised by real-time PCR (qPCR), cell viability assessed by MTT assay and protein expression of caspase-3, apoptotic marker, by immunocytochemistry and subsequent quantification by optical densitometry. After 24 hours of incubation with IL-25 at concentrations of 1, 10 and 50 ng/mL was found a significant decrease in cell viability at 1 ng/mL (38.5 % for MCF-7 cells and 74.0 % for MDA-MB-231 cells; p<0.05) compared with control group, and increased expression of caspase-3 (p<0.001) in both lineage cells. The treatment with siRNA IL-17B at 10nM significantly decreased cell viability (80.0% for MCF-7 cells and 86.0% for MDA-MB-231 cells, p<0.05), and showed a slight increase in the expression of caspase-3. When treated with melatonin at concentrations from 0.001 to 1 mM was observed a significant decreased in cell viability at 1 mM (70.0% for MCF-7 cells and 41.0% for MDA-MB-231 cells; p<0.05) and high expression of caspase-3 (p<0.001) in both lineage cells. For MCF-10A cells, there was no decrease in cell viability to treatments proposed, enabling the use of these therapeutic agents. The joint treatments showed synergic action in reducing the cell viability and induction apoptosis. Our results reinforce the anti-proliferative properties of these agents in breast cancer, establishing new therapeutic strategies. In addition, understanding the role of these factors in immune modulating highlights the growing connection between the contribution of the immune system to combat cancer progression.

## Financial support

FAPESP

